# Autophagy inhibition sensitizes LY3023414-induced anti-glioma cell activity *in vitro* and *in vivo*

**DOI:** 10.18632/oncotarget.22147

**Published:** 2017-10-27

**Authors:** Lan Zheng, Huanyin Li, Yanqing Mo, Gong Qi, Bin Liu, Jing Zhao

**Affiliations:** ^1^ Neurology Department, Minhang Hospital, Fudan University, Shanghai, China

**Keywords:** glioma, PI3K-AKT-mTOR, LY3023414, autophagy, apoptosis

## Abstract

PI3K-AKT-mTOR signaling is a valuable treatment target for human glioma. LY3023414 is a novel, highly-potent and pan PI3K-AKT-mTOR inhibitor. Here, we show that LY3023414 efficiently inhibited survival and proliferation of primary and established human glioma cells. Meanwhile, apoptosis activation was observed in LY3023414-treated glioma cells. LY3023414 blocked AKT-mTOR activation in human glioma cells. Further studies show that LY3023414 induced feedback activation of autophagy in U251MG cells. On the other hand, autophagy inhibition via adding pharmacological inhibitors or silencing Beclin-1/ATG-5 significantly potentiated LY3023414-induced glioma cell apoptosis. *In vivo* studies demonstrated that U251MG xenograft tumor growth in mice was suppressed by oral administration of LY3023414. Remarkably, LY3023414's anti-tumor activity was further augmented against the Beclin-1-silenced U251MG tumors. Together, our results suggest that targeting PI3K-AKT-mTOR cascade by LY3023414 inhibits human glioma cell growth *in vitro* and *in vivo*. Autophagy inhibition could further sensitize LY3023414 against human glioma cells.

## INTRODUCTION

Malignant glioma is the common primary tumor in central nervous system (CNS) [1–4]. It is estimated that over 18,000 cases of glioma will be diagnosed each year in the US alone [5, 6]. The prognosis of human glioma is among the worst of all malignancies, and the treatment option is very limited [1–4]. The overall-survival of malignant glioma is only 6-12 months [5, 6]. Therefore, it is needed to develop novel therapeutic strategies to treat human glioma [1–4].

The PI3K (phosphoinositide 3-kinase)-AKT-mTOR (mammalian target of rapamycin) cascade is a key oncogenic pathway in human malignancy [7–9]. Dysregulation of this pathway is often detected in human glioma, which is associated with tumor transformation, tumorigenesis and progression [10–15]. Over-activation of PI3K-AKT-mTOR in glioma is vital for many cancerous behaviors [7–9, 11, 16, 17]. Thus, PI3K-AKT-mTOR cascade is important therapeutic target for human glioma [10–14] and many other malignancies [8, 9]. Recent studies have characterized a novel, highly-potent and pan PI3K-AKT-mTOR inhibitor, named LY3023414. The potential activity of this inhibitor against human glioma cells was tested in this study.

Autophagy is mainly a pro-survival response in cancer [18–20]. Autophagy starts with the formation of autophagosomes, where certain cytoplasm components are enclosed into the double-membrane structure [18–20]. Lysosomes were then fused with autophagosomes to digest the enclosed components [18–20]. This process is known to provide nutrients for cell survival [18–20]. A number of cancer-killing agents can induce feedback activation of autophagy, which attenuates anti-cancer cell activity by these agents [21–25]. In the current study, we show that autophagy activation could be an important resistance factor of LY3023414 in glioma cells. Autophagy inhibition sensitizes LY3023414-induced activity against human glioma cells.

## RESULTS

### LY3023414 inhibits human glioma cell survival and proliferation

**Figure 1:**
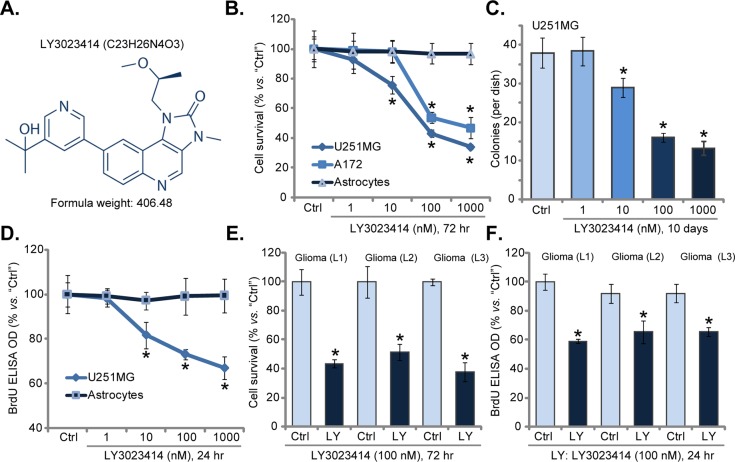
LY3023414 inhibits human glioma cell survival and proliferation The structure and formula weight of LY3023414 were presented **(A)**. Established human glioma cells (U251MG and A172 lines), primary human astrocytes (“Astrocytes”) or primary human glioma cells [three lines, “Glioma (L1/2/3)”], were either left untreated (“Ctrl”) or treated with LY3023414 at 1-1000 nM, cells were further cultured for indicated time; Cell survival was tested (**B**, **C**, and **E**) (n=5). Cell proliferation was also tested by the BrdU ELISA assay (**D** and **F**) (n=5). ^*^p < 0.05 *vs*. “Ctrl”. Experiments in this figure were repeated three times.

In order to study the potential effect of LY3023414 (its structure is presented in Figure 1A), established human glioma cell lines, U251MG and A172, were treated with LY3023414. CCK-8 survival assay results in Figure 1B demonstrated that LY3023414 treatment dose-dependently reduced CCK-8 OD of U251MG cells and A172 cells, displaying anti-survival/cytotoxic activity. On the other hand, LY3023414 treatment was non-cytotoxic to primary human astrocytes (Figure 1B). Colony formation assay results in Figure 1C demonstrated that LY3023414, at 10-1000 nM, significantly decreased number of viable U251MG colonies, further confirming its anti-survival activity. Next, BrdU ELISA assay was applied to test cell proliferation. Results showed that LY3023414 dose-dependently inhibited U251MG cell proliferation (BrdU ELISA OD, Figure 1D). It was yet again in-effective to proliferation of normal primary human astrocytes (Figure 1D).

Three primary human glioma cell lines were established, named as “Glioma (L1/L2/L3)”. These primary cancer cells were also treated with LY3023414. Results from CCK-8 survival assay (Figure 1E) and BrdU proliferation assay (Figure 1F) demonstrated that LY3023414 (100 nM) was cytotoxic and anti-proliferative to all three primary glioma cell lines. Notably, for testing cell proliferation, cells were only treated with LY3023414 for only 24 hours, thus making sure no cell death had occurred. Together, these results confirm that LY3023414 inhibits human glioma cell survival and proliferation.

### LY3023414 provokes apoptosis in human glioma cells

**Figure 2:**
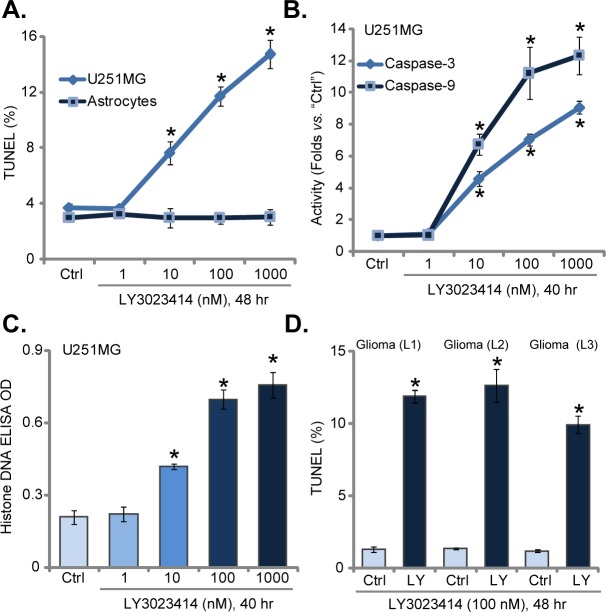
LY3023414 provokes apoptosis in human glioma cells U251MG cells, primary human astrocytes (“Astrocytes”) or primary human glioma cells [“Glioma (L1/2/3)”], were either left untreated (“Ctrl”) or treated with LY3023414 (at 1-1000 nM), cells were further cultured for indicated time. Cell apoptosis was tested by the listed assays (**A-D**) (n=5). ^*^p < 0.05 *vs*. “Ctrl”. Experiments in this figure were repeated three times.

Inhibition of cell survival/proliferation could be due to apoptosis induction, we thus tested LY3023414's activity on glioma cell apoptosis. Results in Figure 2A demonstrated that LY3023414 treatment in U251MG glioma cells increased the number of TUNEL-positive cells, suggesting apoptosis activation. LY3023414 again displayed dose-dependent effect in inducing U251MG cell apoptosis (Figure 2A). Yet, same LY3023414 treatment failed to provoke significant apoptosis (TUNEL assay) in primary human astrocytes (Figure 2A). Results in Figure 2B demonstrated that LY3023414 (at 10-1000 nM) significantly increased the activity of caspase-3 and caspase-9 in U251MG cells. Further, LY3023414 treatment also increased the Histone DNA apoptosis ELISA OD (Figure 2C). These results clearly implied that LY3023414 activated apoptosis in U251MG cells. The pro-apoptosis activity by LY3023414 was again dose-dependent (Figure 2B–2C). TUNEL nuclei staining assay results in Figure 2D confirmed that LY3023414 also induced apoptosis in all three lines of primary glioma cells. The percentage of TUNEL nuclei was increased after LY3023414 (100 nM, 48 hours) treatment (Figure 2D). Therefore, LY3023414 provokes apoptosis in human glioma cells.

### LY3023414 blocks AKT-mTOR activation in human glioma cells

**Figure 3:**
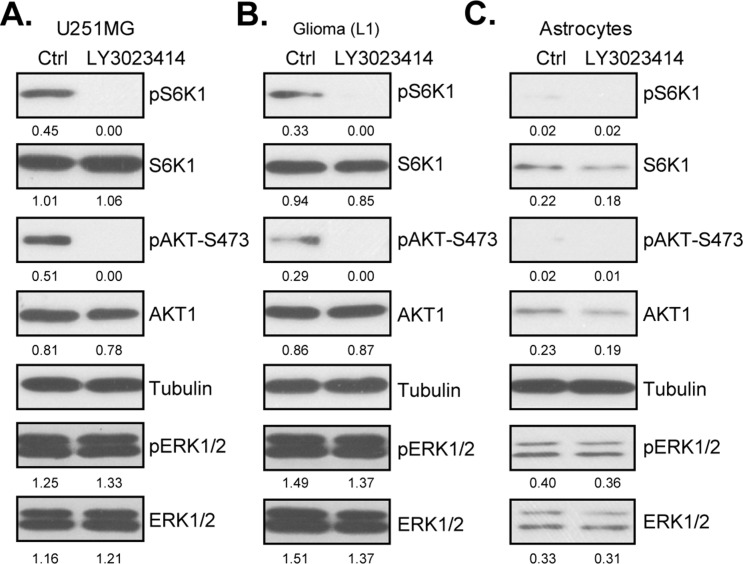
LY3023414 blocks AKT-mTOR activation in human glioma cells U251MG cells **(A)**, primary human glioma cells [“Glioma (L1)”] **(B)** or primary human astrocytes (“Astrocytes”) **(C)** were either left untreated (“Ctrl”) or treated with LY3023414 (100 nM) for 1 hour, expressions of listed proteins were tested by Western blotting assay. Expressions of indicated proteins were quantified (total gray, *vs*. Tubulin). Experiments in this figure were repeated three times.

Dysregulation of PI3K-AKT-mTOR signaling is imporant in glioma tumorigenesis and progression, which is important for cell survival, proliferation and apoptosis resistance. LY3023414 is a newly identified pan PI3K-AKT-mTOR inhibitor [26–29]. We then wanted to know if LY3023414 could also block PI3K-AKT-mTOR signaling in glioma cells. Western blotting assay results in Figure 3A showed that treatment with LY3023414 (100 nM, 1 hour) in U251MG cells almost completely blocked phosphorylated (“p”) AKT (Ser-473) and pS6K1 (Thr-389). pERK1/2, another key oncogenic signaling, was unchanged by the LY3023414 treatment (Figure 3A). Expressions of total AKT, S6K1 and ERK1/2 were also not changed after LY3023414 treatment (Figure 3A, quantification). Very similar results were also observed in primary human glioma cells [“Glioma (L1)”], where LY3023414 largely inhibited activation of AKT-mTOR, but not ERK (Figure 3B). Intriguingly, basal level of AKT-mTOR activation (pAKT/pS6K1), as well as total AKT and S6K1, were much lower in the primary human astrocytes (Figure 3C, quantification). This could possibly explain why these cells were not targeted by LY3023414 (Figures 1 and 2). Notably, ERK activation and expression was also much lower in primary astrocytes (Figure 3C). Collectively, these results demonstrate that LY3023414 blocks AKT-mTOR activation in human glioma cells.

### LY3023414 induces autophagy activation in glioma cells

**Figure 4:**
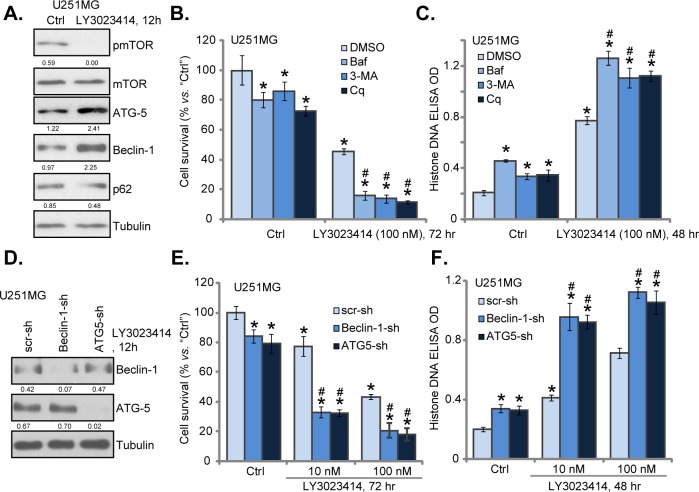
LY3023414 induces autophagy activation in U251MG cells U251MG cells were treated with LY3023414 (100 nM) for 12 hours, expressions of listed proteins were tested by Western blotting assay **(A)**. U251MG cells were pretreated with 3-methyladenine (3-MA, 10 mM), bafilomycin A1 (Baf A1, 1 μM) or chloroquine (Cq, 100 μM) for 1 hour, followed by LY3023414 (10/100 nM) treatment, cells were further cultured for indicated time. Cell survival **(B)** and apoptosis **(C)** were tested (n=5). Stable U251MG cells, expressing lentiviral Beclin-1 shRNA (“Beclin-1-sh”), ATG-5 shRNA (“ATG5-sh”) or scramble control shRNA (“scr-sh”), were treated with LY3023414 (100 nM), cells were further cultured for indicated time. Expressions of listed proteins were shown **(D)**. Cell survival **(E)** and apoptosis **(F)** were tested similarly (n=5). Expressions of indicated proteins were quantified (total gray, *vs*. Tubulin) (A and D). ^*^p < 0.05 *vs*. “Ctrl”. ^#^ *p* < 0.05 *vs*. LY3023414 treatment of “DMSO” (0.1 %) (B and C) or “scr-sh” (D and E). Experiments in this figure were repeated three times.

The other important focus of this study is to identify possible resistance factor of LY3023414. Existing evidences have implied that AKT-mTOR inhibition in cancer cells could lead to feedback activation of autophagy, which counteracts cancer cell apoptosis [21–25]. On the other hand, autophagy inhibition could sensitize or potentiate the anti-cancer activity by AKT-mTOR inhibitors [21–25]. Western blotting assay results in Figure 4A suggested that LY3023414 treatment in U251MG cells possibly also provoked autophagy, which was evidenced by mTOR inactivation, Beclin-1 and ATG-5 induction, as well as p62 downregulation (Figure 4A) [21, 22, 30–32].

Next, various autophagy inhibitors of different mechanisms of action were applied, which included 3-methyladenine (3-MA) [33], chloroquine (Cq) [34] and bafilomycin A1 (Baf A1) [35]. As demonstrated, co-treatment with these autophagy inhibitors dramatically augmented LY3023414 (100 nM)-induced U251MG cell death (Figure 4B) and apoptosis (Figure 4C). In other words, pharmacological inhibition of autophagy sensitized LY3023414-induced anti-glioma cell activity (Figure 4B and 4C). Notably, the autophagy inhibitors alone also induced minor viability reduction and apoptosis in U251MG cells (Figure 4B and 4C), suggesting that basal autophagy induction could also be pro-survival or anti-apoptotic.

Genetic strategy was then applied. Lentiviral Beclin-1 shRNA or ATG-5 shRNA was added to U251MG cells to. As shown in Figure 4D, Beclin-1 or ATG-5 expression was largely downregulated by the targeted shRNA in stable U251MG cells. Remarkably, LY3023414-induced cell death (Figure 4E) and apoptosis (Figure 4F) were significantly potentiated in Beclin-1-/ATG-5-silenced U251MG cells. Thus, silencing Beclin-1 or ATG-5 could also efficiently sensitize LY3023414's activity in glioma cells. These results again suggest that autophagy inhibition could sensitize LY3023414 in human glioma cells. Notably, U251MG cells with Beclin-1/ATG-5 shRNA showed slightly decreased cell survival and minor apoptosis (Figure 4E and 4F).

### LY3023414 administration inhibits U251MG tumor growth in mice

**Figure 5:**
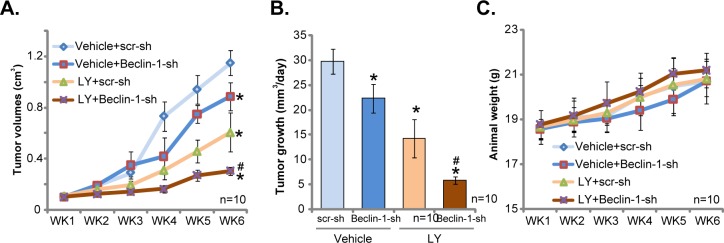
LY3023414 administration inhibits U251MG tumor growth in SCID mice SCID mice bearing U251MG tumor with lentiviral Beclin-1 shRNA (“Beclin-1-sh”) or scramble control shRNA (“scr-sh”) were treated with vehicle control (“Vehicle”) or LY3023414 (“LY”, gavage, 25 mg/kg, daily, 14 consecutive days). Tumor volumes (**A**, in cm^3^) and mice body weights (**C**, in grams) were recorded weekly for 5 total weeks. Estimated daily tumor growth, in mm^3^ per day, was also calculated **(B)**. ^*^p < 0.05 *vs*. “Vehicle+scr-sh” group. ^#^ *p* < 0.05 *vs*. “LY+scr-sh” group. n=10 mice per group.

At last, the activity of LY3023414 *in vivo* was tested. As discussed, stable U251MG cells, expressing lentiviral Beclin-1 shRNA (“Beclin-1-sh”) or scramble shRNA (“scr-sh”) were inoculated via *s.c*. injection to the severe combined immuno-deficient (SCID) mice. Within three weeks, the U251MG tumor reach 100 mm^3^ in volume. Mice were then treated with LY3023414, which was given daily by gavage at 25 mg/kg body weight (for 14 consecutive days). Half of the mice were treated with vehicle control. Tumor volume curve results in Figure 5A demonstrated that treatment of LY3023414 significantly inhibited growth of scr-sh-expressing U251MG tumors in SCID mice. Remarkably, LY3023414's anti-tumor activity *in vivo* was further augmented against Beclin-1-sh-expressing tumors (Figure 5A). These results imply that autophagy inhibition (via silencing Beclin-1) should also sensitize LY3023414's activity *in vivo*. Indeed, estimated daily tumor growth results in Figure 5B further confirmed the above results. Daily tumor growth, in mm^3^ per day, was lowest in LY3023414-treated tumors with Beclin-1 shRNA (Figure 5B). Notably, growth of Beclin-1-sh-expressing tumors (vehicle treatment) was also slightly inhibited, as compared to control tumors (Figure 5A and 5B). Results in Figure 5C showed that the mice body weights were not significantly different between the groups, indicating that the above-mentioned treatment regimens were relatively safe to the experimental animals.

## DISCUSSION

PI3K-AKT-mTOR cascade is often dysregulated in glioma cells, leading to constitutive activation [11, 36]. This signaling is associated with glioma tumorigenesis and progression [11, 36]. We here show that LY3023414, a novel and pan PI3K-AKT-mTOR inhibitor [26–29], potently inhibited survival and proliferation of established and primary human glioma cells. *In vivo* studies found that oral administration of LY3023414 inhibited U251MG xenograft tumor growth in the nude mice. Intriguingly, treatment with LY3023414 was safe to non-cancerous primary human astrocytes. LY3023414 administration in mice also didn't induce apparent toxicities. Therefore, it will be interesting to further test LY3023414 as a novel anti-glioma agent.

Autophagy is mainly a cytoprotective process, where cell degrades its own components via lysosomal machineries [37]. It is tightly regulated by the actions of various kinases including AKT-mTOR [38, 39]. For instance, mTOR activation shuts down autophagy via directly inhibiting ULK1 pathway [40]. mTOR inhibition, on the other hand, could then lead to feedback activation of autophagy [38, 39], which reportedly counteracts the anti-cancer activity by a number of mTOR inhibitors [21]. In the current study, we showed that LY3023414 blocked AKT-mTOR activation in glioma cells, also causing feedback activation of autophagy. The latter was evidenced by mTOR in-activation, Beclin-1/ATG-5 upregulation, and p62 degradation [41].

The novel finding of the current study is that inhibition of autophagy, via genetic or pharmacologic strategies, significantly sensitized LY3023414-induced killing of glioma cells *in vitro* and *in vivo*. Indeed, recent literatures have implied that blockage of autophagy could potentiate the anti-cancer activity by a number of PI3K-AKT-mTOR inhibitors [21, 42, 43]. Chloroquine (Cq), the well-established autophagy inhibitor, increases intra-lysosomal pH, and shuts down the lysosomal degradation pathway [44]. Another well-established autophagy inhibitor, 3-methyladenine (3-MA), mainly interferes LC3B-I-LC3B-II conversion, therefore preventing autophagosome formation [33]. Bafilomycin A1 (Baf A1) is also a known autophagy inhibitor, which inhibits fusion between autophagosome and lysosome [35]. These autophagy inhibitors of different mechanism of actions all potentiated LY3023414-induced glioma cell apoptosis, confirming the pivotal function of autophagy in LY3023414 resistance.

Besides these pharmacological evidences, our results further showed that silencing Beclin-1/ATG-5 also significantly potentiated LY3023414-induced glioma cell death and apoptosis. Further, LY3023414's anti-tumor activity was augmented against Beclin-1-silenced tumors. Both Beclin-1 and ATG-5 are key autophagic regulators [32, 45]. These genetic evidences once again support the negative function of autophagy in LY3023414-mediated anti-glioma cell activity.

## MATERIALS AND METHODS

### Chemicals and reagents

LY3023414 was provided by Selleck (Beijing, China). Antibodies of this study were purchased from Cell Signaling Technology (Wuhan, China). The autophagy inhibitors, 3-methyladenine (3-MA), chloroquine (Cq) and bafilomycin A1 (Baf A1) were obtained from Sigma-Aldrich (Shanghai, China). The cell culture reagents and antibiotics were obtained from Hyclone (Shanghai, China).

### Culture of established human glioma cells

The human glioma cell lines, A172 and U251MG, were provided by the Cell Bank of Institution of Biological Science of China (Shanghai, China). Cells were cultured in DMEM with FBS (10%) and necessary antibiotics.

### Culture of primary human glioma cells

Three written-informed primary glioma patients (53/64/62 years old, male, stage III) were enrolled. The patients received no radiation or chemotherapy prior to surgery. Fresh tumor tissues were minced, followed by digestion. The resulting primary cells were filtered via 70-μm nylon cell strainer. Primary cancer cells were maintained in described complete DMEM/F12 medium [10]. A total of three lines of primary glioma cells [“Glioma (L1/2/3)”] were established. The protocols using human samples/cells were in accordance with the principles expressed in the Declaration of Helsinki, and were approved by the Institutional Review Board (IRB) and Ethics Board of Fudan University. Written-informed consent was obtained each participant.

### Primary culture of human astrocytes

Human primary astrocyte cultures were provided by the Cell Bank of Fudan University (Shanghai, China) [10]. Over 99% of astrocytes were GFAP (glial fibrillary acidic protein) positive. The primary astrocytes were maintained under the astrocyte media described previously [10].

### CCK-8 assay of cell proliferation

Cells were initially seeded onto 96-well plates at 1 ×10 ^4^ cells/well. After treatment, cell survival was measured by the Cell Counting Kit-8 (CCK-8) (Dojindo, Japan) assay. The optical density (OD) value of CCK-8 was recorded.

### Colony formation assay

U251MG cells (1 ×10^5^ per dish) with the LY3023414 treatment were trypsinized and suspended in 1 mL medium plus 1 % agarose (Sigma). Cells were then plated on the 10-cm culture dish (pre-solidified with agarose). After 10 days of incubation, U251MG colonies were stained, and the number of colonies was recorded.

### Western blotting assay

RIPA lysis buffer (Biyuntian, Nanjing, China) was applied to achieve cell or tissue protein lysates. The protein concentration was determined by the Bio-Rad reagents (Bio-Rad, Shanghai, China). Aliquots of 30 μg protein lysates per condition were separated by 10-12% of SDS-PAGE gels, followed by transferring to the PVDF membranes (Millipore, Suzhou, China). Afterward blocking in 10% milk, the blots were probed with indicated primary and secondary antibodies. Afterward, the ECL reagents (GE, Shanghai, China) were applied to visualize the targeted protein bands. Band intensity was always quantified via the ImageJ software (NIH), and the value was normalized to the loading control [46].

### BrdU ELISA assay

Cells with the LY3023414 treatment were further incubated with BrdU dye (10 μM, Cell Signaling Tech, Shanghai, China). BrdU incorporation in the glioma cells was assayed via the BrdU ELISA kit (Cell Signaling Tech) with manufactory's protocol. ELISA OD at 405 nm was recorded.

### TUNEL staining

After the applied LY3023414 treatment, cell nuclei were stained with the TUNEL fluorescence dye (Sigma, Shanghai, China). TUNEL fluorescence was then viewed under the Leica Confocal microscope. TUNEL positive nuclei ratio (*vs*. total cell nuclei, stained with Hoechst) was recorded. For each condition, at least 200 nuclei were counted.

### Histone DNA apoptosis ELISA analysis

Apoptosis in LY3023414-treated cells was also quantified via the Histone DNA Apoptosis ELISA Kit (Roche, Beijing, CA). Detailed protocol was described in previous studies [47–49].

### Caspase activity assay

Cytosolic protein extracts (30 μg of each condtion) were added to the caspase assay buffer [50], along with caspase-3 substrate [DEVD-7-amido-4-(trifluoromethyl)-coumarin (AFC)] or the caspase-9 substrate (LEHD-AFC). The AFC intensity OD was tested by a microplate reader with excitation value of 400 nm and emission value of 550 nm.

### shRNA

The lentiviral Beclin-1 shRNA was obtained from Santa Cruz Biotech (sc-29797-V). The lentiviral ATG-5 (Autophagy protein 5) shRNA was provided by Dr. Jiang's group [32]. The lentiviral shRNA (10 μL/mL medium) was added to U251MG cells for 24 hours. Cells were then selected by puromycin (2.5 μg/mL) for 8-10 days. Control cells were incubated with same amount of lentiviral scramble control shRNA (sc-108080-V, Santa Cruz). Beclin-1 and ATG-5 protein expression in stable cells was tested by Western blotting assay.

### Mice xenograft assay

Stable U251MG cells (1 × 10 ^7^ cells per mouse in Matrigel), with lentiviral Beclin-1 shRNA or scramble shRNA, were subcutaneously (*s.c*.) injected to the flanks of female severe combined immuno-deficient (SCID) mice. Mice were randomized into four groups with 10 mice per group. Treatment was initiated with vehicle or LY3023414 (25 mg/kg daily, gavage) when established tumors were ~100 mm^3^ in volume. Tumor volume and mouse body weight were recorded weekly. Tumor volumes were calculated via the formula: (cm^3^) = (the shortest diameter^2^ × the longest diameter)/2. Animals were observed on daily bases. All efforts were made to minimize suffering. The animal protocols were approved by Institutional Animal Care and Use Committee (IACUC) and Ethics committee of Fudan University.

### Statistics

The results were expressed as the mean ± standard deviation (SD). Statistical significance (*p* < 0.05) was evaluated by one-way ANOVA followed by Bonferroni post hoc test (SPSS 16.0, Chicago, IL).

## CONCLUSIONS

In summary, our results suggest that targeting PI3K-AKT-mTOR cascade by LY3023414 inhibits glioma cell growth *in vitro* and *in vivo*. Autophagy inhibition could be a fine strategy to further sensitize LY3023414's activity against glioma cells.
